# Supplementary figures and images for: EpiFoundation: A Foundation Model for Single-Cell ATAC-seq via Peak-to-Gene Alignment
Source: bioRxiv. 2025 Sep 28:2025.02.05.636688. Originally published 2025 Feb 8. Preprint. [Version 2] doi: 10.1101/2025.02.05.636688 (PMC11839112; doi:10.1101/2025.02.05.636688)

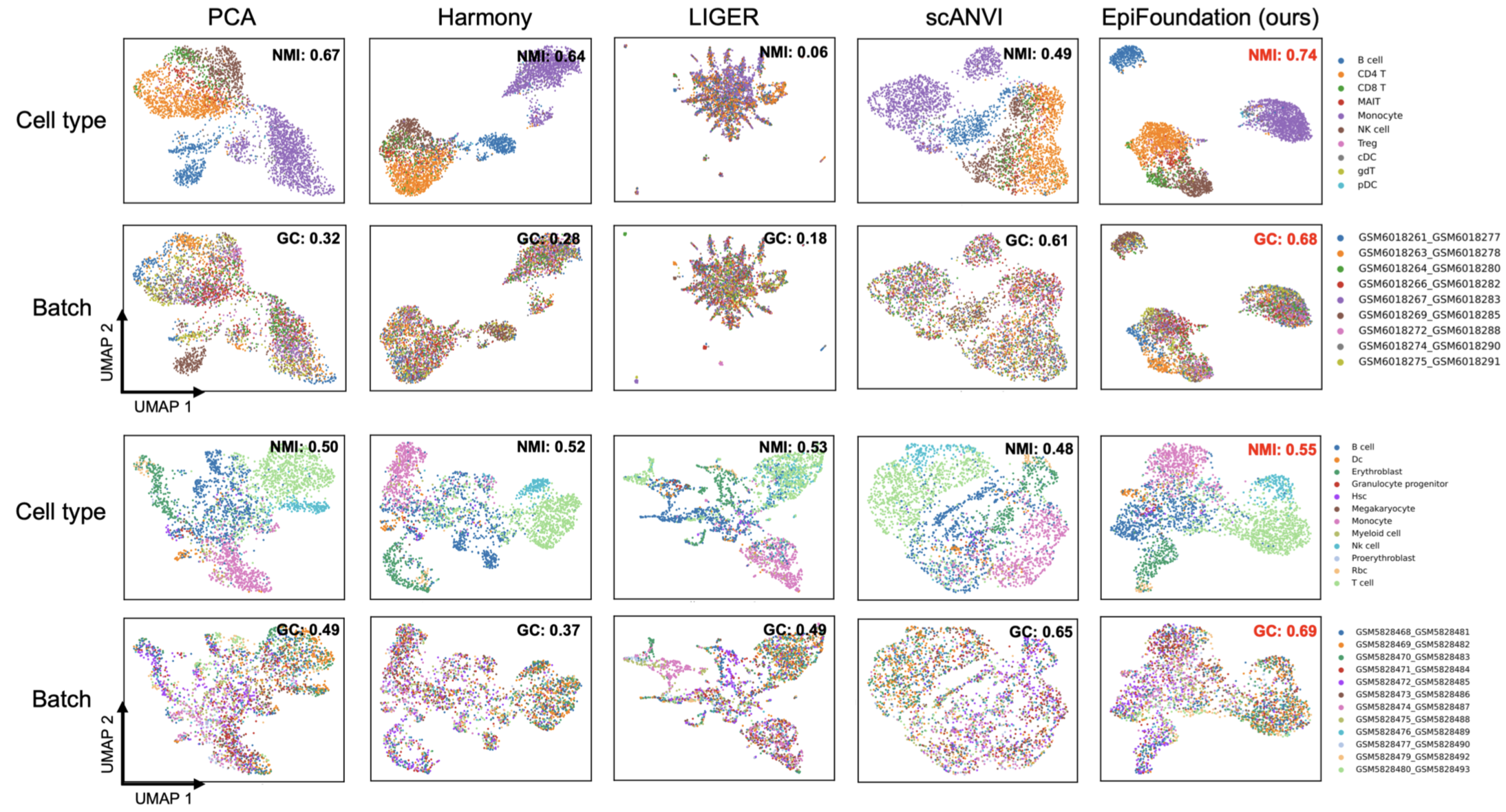

Supplement: Supplement 1 [file media-1.zip › EpiFoundation-main/assets/batch_correlation.png]

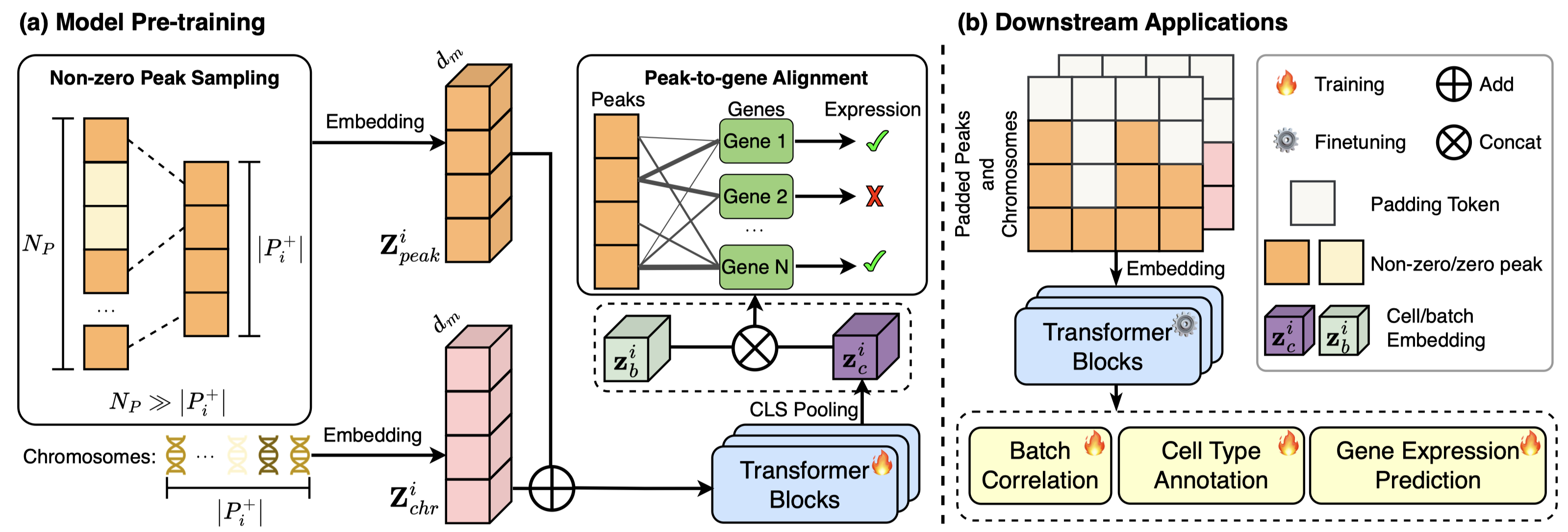

Supplement: Supplement 1 [file media-1.zip › EpiFoundation-main/assets/framework.png]
